# Detection of an oestrogen receptor-like protein in human meningiomas by band shift assay using a synthetic oestrogen responsive element (ERE).

**DOI:** 10.1038/bjc.1993.330

**Published:** 1993-08

**Authors:** S. G. Koehorst, H. M. Jacobs, J. H. Thijssen, M. A. Blankenstein

**Affiliations:** Department of Endocrinology, Academic Hospital Utrecht, The Netherlands.

## Abstract

**Images:**


Br. J. Cancer (1993), 68, 290-294 © Macmillan Press Ltd., 1993

### Detection of an oestrogen receptor-like protein in human meningiomas by band shift assay using a synthetic oestrogen responsive element (ERE)

S.G.A. Koehorst, H.M. Jacobs, J.H.H. Thijssen & M.A. Blankenstein

*Department of Endocrinology, Academic Hospital Utrecht, Utrecht, The Netherlands.*

**Summary** When a ligand binding or enzyme immunoassay is used, meningiomas are found to be rich in progestin receptors (PR) whereas oestrogen receptors (ER) are virtually undetectable. A protein that can bind to an oestrogen responsive element (ERE) was detected in meningiomas, by using the band-shift assay. The binding of ER to the ERE is inhibited by the anti-ER monoclonal antibody ER-P31, which is directed to the A/B domain of the ER, indicating that the binding protein is an ER-like protein.

Female sex hormones may be involved in the etiology of human meningioma. Following the first report in 1979, on the putative presence of oestrogen receptors (ER) in human meningiomas (Donnell *et al.*, 1979), several research groups, including our own (Blankenstein *et al.*, 1983; Martuza *et al.*, 1985) have tried to confirm these findings. Using Scatchard plot analysis (Blaauw *et al.*, 1986) or enzyme immunoassay (Blankenstein *et al.*, 1987) the majority of meningiomas were found to be ER negative. The same experiments revealed that meningiomas contain high amounts of progestin receptors (PR). Since in classical female sex steroid target tissues like breast and uterus, the synthesis of PR is ER dependent the finding that (at the protein level at least) meningiomas are rich in PR, yet virtually devoid of ER initiated further research.

We recently reported on the detection of mRNA coding for the wild type receptor by using the reversed-transcriptase-PCR technique (Koehorst *et al.*, 1992). Apart from the wild type (wt) mRNA we also found two alternatively spliced mRNA's. One variant is missing exon 4 and the other is missing exon 7 (Koehorst *et al.*, 1993).

The present study was designed to evaluate the presence in meningiomas of ER or ER-like proteins which could induce PR synthesis. We hypothesised that if the ER (at concentrations below the detection limit of the conventional receptor assay) or ER-like protein (escaping detection by lack of ligand binding) is responsible for the induction of PR synthesis in meningiomas at least a protein should be present which can bind to the oestrogen responsive element (ERE) of the PR-gene. The aim of the present investigation, therefore, was to test if such an ERE-binding protein is present in meningioma. To this end the binding of meningioma cytosol components to a synthetic ERE was investigated with the band shift assay. This assay is a very sensitive method to detect ERE binding proteins. Scott *et al.* (1991) could detect as little as 0.1 fmol of DNA bound ER from whole cell extracts containing 50 µg protein.

We found evidence for the existence of a protein that binds to the ERE and is recognised by the specific anti-ER antibody ER P31.

### Materials and methods

### *Tissues*

Human meningioma tissue was placed on ice immediately after removal from the patient. Representative specimens were frozen at − 80°C until they were used for cytosol preparation or receptor assay. The MCF7 breast cancer cell line was routinely grown with 10% foetal calf serum (Gibco BRL, Paisely, UK) with RPMI 1640 (Gibco BRL, Paisely, UK). Cells were harvested by scraping with a rubber policeman in PBS.

### *Biochemical assay of steroid hormone binding*

The ER and PR content of the tumours were measured as described previously (Blankenstein *et al.*, 1983) by the ligand binding assay according to the guidelines of the EORTC, Breast Cancer Cooperative Group (EORTC, Breast Cancer Cooperative Group, 1980).

### *Cytosol preparation for ERE-binding experiment*

Approximately 400 mg of fresh tissue powder was added to 1.5 ml of ice cold extraction buffer (0.4 M KCl, 10 mM Tris (pH 7.4), 1 mM EDTA, 10 mM monothioglycerol, 10% glycerol (v/v)) or $20 \times 10^6$ MCF7 cells were dounced in 1.5 ml ice cold extraction buffer. The resulting homogenate was centrifuged for 5 min at 4°C at 3,600 r.p.m. The supernatant was centrifuged again in a swing out rotor at 132,000 ***g*** for 10 min at 4°C. Twenty-five µl of the clear supernatant was used for protein determination by the Coomassie Brilliant Blue method (Biorad, Richmond, CA, USA).

### *Band shift assay*

As probe for the band shift assay, we chose the ERE from the vitellogenin A2 gene which has been shown to have high regulatory potential in transient transfection of ERE-TKCAT reporter construction into oestrogen responsive MCF7 cells (Klein-Hitpass *et al.*, 1986). Both strands corresponding to the ERE were synthesised as 31 (5'-GATCCGTCAGGTCACAGTGACCTGATGGATC-3'; palindrome is underlined) base oligonucleotides using an Applied Biosystems DNA synthesiser (San Jose, CA, USA). Equimolar amounts of the two strands were annealed in buffer (10 mM Tris, 1 mM EDTA (pH 8.0)) by heating to 95°C and cooling to room temperature during a period of 2 h. The double stranded ERE oligomer was end-labelled using [$\delta - {}^{32}P$] ATP (Amersham, Amersham, UK) and T4 polynucleotide kinase (Boehringer Mannheim, Mannheim, Germany). Five µl of the supernatant containing 20–30 µg protein (for MCF7 containing 2 µg of protein) were added to 15 µl incubation buffer (100 mM Tris (pH 8.0), 1 mM EDTA, 10% glycerol (v/v), 2 µg poly(dI.dC)) and placed on ice. After 15 min 1 ng $^{32}P$-labelled double-stranded ERE oligomer was added and the reaction mixture incubated for 20 min at 20°C. The protein-ERE complexes were separated by electrophoresis through 6% acrylamide (19:1, acrylamide;bis) gels using a buffer consisting of 50 mM Tris, 50 mM boric acid, 1 mM EDTA (pH 8.0). The gels were run at 180 V for 3 h, vacuum dried and autoradiographed. The ER-containing human breast cancer cell line MCF7 was used as a positive

Correspondence: S.G.A. Koehorst, Department of Endocrinology G02.625, Academic Hospital Utrecht, PO Box 85500, NL-3508 GA Utrecht, The Netherlands.
Received 7 December 1992; and in revised form 3 March 1993.

control for ERE binding.

To confirm the integrity of the protein isolated from the tumours, a band shift assay was performed using a oligonucleotide which contained the Spl binding consensus sequence (5'-CAAAGTCTGGGCGGGCCGATCAAG-'3; consensus is underlined). For the competition binding analysis in which the specificity of the ERE was proven, a oligonucleotide was used which contained the consensus of the progesterone responsive element (PRE)(5'-CCAAA-GTCAGAACACAGTGTTCTGATCAAG-3'; palindrome is underlined and differences with palindrome of ERE are in bold underlined).

### Results

The ERE from the vitellogenin A2 gene which was used in the band shift assay has proven specificity in oestrogen binding experiments (Klein-Hitpass *et al.*, 1988; 1989; Klock *et al.*, 1987).

The specificity of binding of the ERE oligonucleotides to ER in crude, high salt extracts from MCF7 and meningioma is shown in Figure 1. The intensity of the ER-ERE complex decreases with increasing ratios of unlabelled/labelled ERE, while the intensity of the ER-ERE complex is unaffected with increasing molar excess of PRE (Figure 1a and b). When MCF7 cytosol was run together with the meningioma sample a single, prominent band co-migrating with that obtained from MCF7 whole cell extract was observed. We attribute this result to the presence of ER or an ER-like protein in the meningioma tissue.

To identify the ER in the ER-ERE complex, cytosols were incubated with the anti-ER monoclonal antibody ER-P31 (MEDAC, Hamburg, Germany). This antibody inhibits the binding to the ERE of the ER from MCF7 cells as well as the ER-like protein in the two meningiomas and the ER in solid breast tumours (Figure 2).

After we had proven the specificity of this ERE oligonucleotide under our experimental conditions, we tested 15 meningiomas with different ER and PR concentration. In all meningiomas but one, including the meningiomas which were ER negative in the ligand binding assay, we could detect a protein which can bind to ERE oligonucleotide (Figure 3, upper panel). The ERE-protein complex from the meningiomas co-migrates with the MCF7 ER-ERE complex even in gels run for longer times (data not shown). To check the integrity of the isolated protein all the meningiomas were tested for Spl binding and all except one were found to be positive (Figure 3, lower panel). This meningioma extract also failed to show ERE-binding. We also checked the ER-ERE binding in meningiomas and the normal meninges from two patients. An ER-ERE complex was detectable in meningiomas, the normal meninges were negative for ER-ERE binding. However the normal meninges was also negative for Spl binding and therefore these results do not allow conclusions to be drawn on differences between normal and tumour tissue.

**Figure 1** Binding specificity of the ERE oligonucleotide in crude, high salt extracts from MCF7 cells and meningioma tissue. Competition binding analysis was performed in which extracts from MCF7 cells **a**, and meningioma tissue **b**, were incubated with 1 ng of $^{32}$P-labelled ERE and increasing amounts of unlabelled ERE as specific competitor or unlabelled PRE as nonspecific competitor as reflected in the molar ratio indicated above each lane.

**Figure 2** Anti-ER monoclonal antibody ER-P31 reduces ERE-protein complex in crude, high salt extracts. The extracts were first incubated with 5 μg antibody ER-P31 or nonspecific antibody (anti-C peptide) at 4°C for 18 h. After administration of 1 ng labelled ERE, the mixture was incubated for 30 min at room temperature and separated on a 6% gel. Extracts from MCF7 cells, an ER +/PR + and ER −/PR + meningioma tissue, and ER +/PR + breast cancer were used. The ER and PR levels are given as fmol/mg protein. (F: protein-free ERE).

**Figure 3** Analysis of ERE-binding activity **a**, and Spl-binding activity **b**, of extracts from 13 meningioma tissues; meningioma tissue (M) and normal meninges (A) from two patients (patient 1 and 2); two normal meninges (Arachn.) and MCF7 (C) as control in the far right and left lanes for the mobility shift assay. The ER and PR levels indicated under each lane are given as fmol/mg protein. (F; protein-free ERE). ERE and Spl binding concur, indicating the integrity of MCF7 and most meningioma extracts but not the normal meninges extracts.

### Discussion

The abundant presence of PR in meningioma and the absence of ER measured by ligand binding assay or enzyme immunoassay prompted us to search for the ER or ER-like protein in meningioma using other techniques. We have already detected mRNA coding for the ER in meningioma (Koehorst *et al.*, 1992). Besides the wild type transcript two variants were detected, one missing exon 7 and the other missing exon 4 (Koehorst *et al.*, 1993). If the wt transcript or the variants are responsible for the PR synthesis, we hypothesise that a protein has to be present which can bind to an ERE.

We analysed DNA-binding activity to the ERE in human meningiomas by a gel mobility assay. The promotor regions of the PR gene are under investigation to define the functional ERE in this gene. So far two functional promotor regions have been described to be important for the induction of PR synthesis by ER. These two regions showed oestrogen inducibility in transient co-transfection experiments with vectors expressing the human ER, although no 'classical' ERE was detected in these promotor regions (Kastner *et al.*, 1990). The sequences which are responsible for this ER inducibility are not well defined. Since no classical ERE is present in the promoter region of the PR gene and as the functional ERE in these regions is not well defined we decided to use a 'classical' ERE from the *Xenopus* vitellogenin A2 gene. This element has been shown to be specific in several band-shift experiments performed by other groups (Klein-Hitpass *et al.*, 1989). We used MCF7 cells as a positive control and demonstrated that the ER-ERE complex derived from meningioma extracts co-migrated with the complex obtained from whole cell extracts of MCF7 cells. The anti-ER monoclonal antibody ER-P31 was able to specifically block ER-ERE complex formation, demonstrating the presence of ER in the complex from MCF7 cells, meningiomas and solid breast cancer derived cytosol (Figure 2). This antibody has been used before in immunohistochemistry (Wilkinson *et al.*, 1988). ER-P31 gives intense nuclear staining on frozen sections of breast carcinoma. Binding of the antibody to ER was confirmed by displacement assays of the receptor on a sucrose gradient (Wilkinson *et al.*, 1988; Wong *et al.*, 1991). The epitope which is recognised by ER-P31 is located in the A/B domain of the receptor. Since this is the variable part of the receptor recognition of ERR-1 or ERR-2 is very unlikely. This antibody inhibits the DNA binding activity of ER. Therefore in the present experiments incubation with antibody was carried out first, followed by incubation with the ERE. If incubation with the ERE is performed first, the antibody hardly interferes with DNA binding of ER. Following binding to the ERE the epitope on the ER is probably no longer accessible to the antibody. Based on the considerations above, we conclude that the ERE used is specific.

We tested 15 meningiomas for the presence of proteins binding to the ERE. The receptor phenotype of these meningiomas ranged from ER/PR positive, ER-negative/PR-positive to ER/PR negative. All but one of the meningiomas were positive for ER-ERE binding, including the ER/PR negative meningiomas (Figure 3, upper panel). This suggests that the protein detected is by itself not sufficient to induce PR synthesis in meningiomas.

It is tempting to speculate that this ER or ER-like protein plays a role in the development and growth of the meningioma and biosynthesis of PR. To test the tumour specificity of the ERE binding, meningiomas and normal meninges from two patients were used. The two meningiomas were positive for ER-ERE binding and also positive for Sp1 binding (Figure 3). The normal meninges however were negative for both. This can be attributed to the fact that normal meningeal tissue is a quiescent tissue. This is confirmed by the fact that we also were not able to isolate any intact mRNA from the normal meninges. Thus the normal meninges cannot be used as a control in this assay and no conclusions can be drawn about the tumour specificity of the ER-ERE binding.

The band shift assay results shown here apparently are in disagreement with those from the ligand binding assay obtained in the past. From experiments with breast tumours it is known however that the band shift assay is not always in agreement with the ligand binding assay. Foster *et al.* (1991) tested 79 breast tumours of which 55 showed that the band shift assay was in agreement with the hormone-binding assay. In 13 tumours the hormone binding assay was negative whereas the band shift assay was positive. In two cases a ER-negative/PR-positive tumour was tested, in both cases the band shift assay was positive (Foster *et al.*, 1991). These two cases have resemblance with the phenotype of meningiomas which are mostly also ER-negative/PR-positive.

In meningiomas an ER or ER-like protein is detectable by the band shift assay but not detectable by ligand-binding (Blaauw *et al.*, 1986) or by enzyme immunoassay (Blankenstein *et al.*, 1987). This may be due to the sensitivity of the assay (Scott *et al.*, 1991).

Another explanation could be a mutation in the hormone-binding domain of the ER so that such an ER variant would not be detected by the ligand binding assay and probably also not in the immunoassay. For example in ER−/PR+ breast tumours an ER variant was detected missing exon 5. This variant was constitutively active in a trans-activation assay (Fuqua *et al.*, 1991). We detected in meningiomas two variant ER mRNA species (Koehorst *et al.*, 1993). One variant with a major deletion in the ligand binding domain was an alternatively spliced product missing exon 7. This variant has no hormone independent transcriptional activity as shown by McGuire *et al.* (1991). Therefore this variant can probably not account for the apparently autonomous PR synthesis in meningiomas. The variant missing exon 4 codes for ER missing amino acid 254–365. Exon 4 includes the last part of the DNA binding domain, the hinge region and the first hundred amino acids of the ligand binding domain. The two zinc fingers of the DNA binding domain are intact so DNA binding can probably occur but no oestradiol will be bound by this variant because a great part of the ligand binding domain is missing. In addition, the highly positively charged region situated between amino acid 251 and 271 is missing. This sequence is very important for the formation of the non-DNA binding 8–9 S ER complexes, which bind heat shock protein 90 (hsp 90) (Chambraud *et al.*, 1990). A variant missing amino acids 251–271 such as the variant missing exon 4 can probably not bind hsp 90 and therefore is always in the 4–5 S DNA binding form. This variant could play a role but whether it is capable of transactivation as well as its relationship to the ER-like protein described here remain to be investigated.

The authors gratefully acknowledge Dr J.W. van't Verlaat, Department of Neurosurgery, Academic Hospital Utrecht and Dr G.H. Blaauw, Department of Neurosurgery, De Wever Hospital, Heerlen, The Netherlands, for making tissues available.

### References

BLAAUW, G., BLANKENSTEIN, M.A. & LAMBERTS, S.W.J. (1986). Sex steroid receptors in human meningioma. *Acta Neurochirgica*, **79**, 42–46.

BLANKENSTEIN, M.A., BLAAUW, G., LAMBERTS, S.W.J. & MULDER, E. (1983). Presence of progesterone receptors and absence of oestrogen receptors in human intracranial meningioma cytosols. *Eur. J. Cancer Clin. Oncol.*, **19**, 365–370.

BLANKENSTEIN, M.A., VAN DER MEULEN-DIJK, C. & THIJSSEN, J.H.H. (1987). Assay of oestrogen and progestin receptors in human meningioma cytosols using immunological methods. *Clin. Chim. Acta*, **165**, 189–195.

CHAMBAUD, B., BERRY, M., REDEUILH, G., CHAMBON, P. & BAULIEU, E.E. (1990). Several regions of human oestrogen receptor are involved in the formation of receptor-heat shock protein 90 complex. *J. Biol. Chem.*, **265**, 20686–20691.

DONNELL, M.S., MEYER, G.A. & DONEGAN, W.L. (1979). Oestrogen receptor protein in intracranial meningiomas. *J. Neurosurg.*, **50**, 499–502.

EORTC. BREAST CANCER COOPERATIVE GROUP. (1980). Revision of the standard for the assessment of hormone receptors in human breast cancer. *Eur. J. Cancer*, **16**, 1513–1515.

FOSTER, B.D., CAVENER, D.R. & PARL, F.F. (1991). Binding analysis of the oestrogen receptor to its specific DNA target site in human breast cancer. *Cancer Res.*, **51**, 3405–3410.

FUQUA, S.A., FITZGERALD, S.D., CHAMNESS, G.C., TANDOM, A.K., MCDONNELL, D.P., NAWAZ, Z., O'MALLEY, B.W. & MCGUIRE, W.L. (1991). Variant human breast tumour oestrogen receptor with constitutive transcriptional activity. *Cancer Res.*, **51**, 105–109.

KASTNER, P., KRUST, A., TURCOTTE, B., STROPP, U., TORA, L., GRONEMEYER, H. & CHAMBON, P. (1990). Two distinct oestrogen regulated promoters generate transcripts encoding the two functionally different human progesterone receptors forms A and B. *EMBO J.*, **9**, 1603–1614.

KLEIN-HITPASS, L., SCHORP, M., WAGNER, U. & RYFFEL, G.U. (1986). An oestrogen-responsive element derived from the 5'-flanking region of the *Xenopus* vitellogenin A2 gene functions in transfected human cells. *Cell*, **46**, 1053-1061.

KLEIN-HITPASS, L., RYFFEL, G.U., HEITLINGER, E. & CATO, A.C.B. (1988). A 13 bp palindrome is a functional oestrogen responsive element and interacts specifically with oestrogen receptors. *Nucleic Acids Res.*, **16**, 647-663.

KLEIN-HITPASS, L., TSAI, S.Y., GREENE, G.L., CLARK, J.H., TSAI, M. & O'MALLEY, B.W. (1989). Specific binding of oestrogen receptor to the oestrogen response element. *Mol. Cell Biol.*, **9**, 43-49.

KLOCK, G., STRAHLE, U. & SCHUTZ, G. (1987). Oestrogen and glucocorticoid responsive elements are closely related but distinct. *Nature*, **329**, 734-736.

KOEHORST, S.G.A., JACOBS, H.M., TILANUS, M.G.J., BOUWENS, A.G.M., THIJSSEN, J.H.H. & BLANKENSTEIN, M.A. (1992). Abberant oestrogen receptor species in human meningioma tissue. *J. Steroid Biochem. Molec. Biol.*, **43**, 57-61.

KOEHORST, S.G.A., JACOBS, H.M., THIJSSEN, J.H.H. & BLANKENSTEIN, M.A. (1993). Wild type and alternatively spliced oestrogen receptor messenger RNA in human meningioma tissue and MCF7 breast cancer cells. *J. Steroid Biochem. Molec. Biol.*, **45**, 227-233.

MARTUZA, R.L., MILLER, D.C. & MACLAUGHLIN, D.T. (1985). Oestrogen and progestin binding by cytosolic and nuclear fractions of human meningiomas. *J. Neurosurg.*, **62**, 750-756.

MCGUIRE, W.I., CHAMNESS, G.C. & FUQUA, S.A.W. (1991). Oestrogen receptor variants in clinical breast cancer. *Mol. Endocrinol.*, **5**, 1571-1577.

SCOTT, G.K., KUSHNER, P., VIGNE, J. & BENZ, C.C. (1991). Truncated forms of DNA-binding oestrogen receptors in human breast cancer. *J. Clin. Invest.*, **88**, 700-706.

WILKINSON, L., ANGÚS, B., AKIBA, R., CORBETT, I., NICHOLSON, S., WESTLEY, B.R. & HORNE, C.H.W. (1988). ER-P31, a new monoclonal antibody recognising the oestrogen receptor effective for immunohistochemistry. *J. Path.*, **155**, 349A.

WONG, S.Y., PURDIE, A., SEWELL, H.F., WILKINSON, L., ANGUS, B., WESTLEY, B. & HORNE, C.H. (1991). Immunohistochemical assessment of ER-P31, a mouse anti-oestrogen receptor protein monoclonal antibody in human breast cancers: comparison with ER-ICA (Abbott) and radio ligand assay. *Tumour Biol.*, **12**, 16-23.


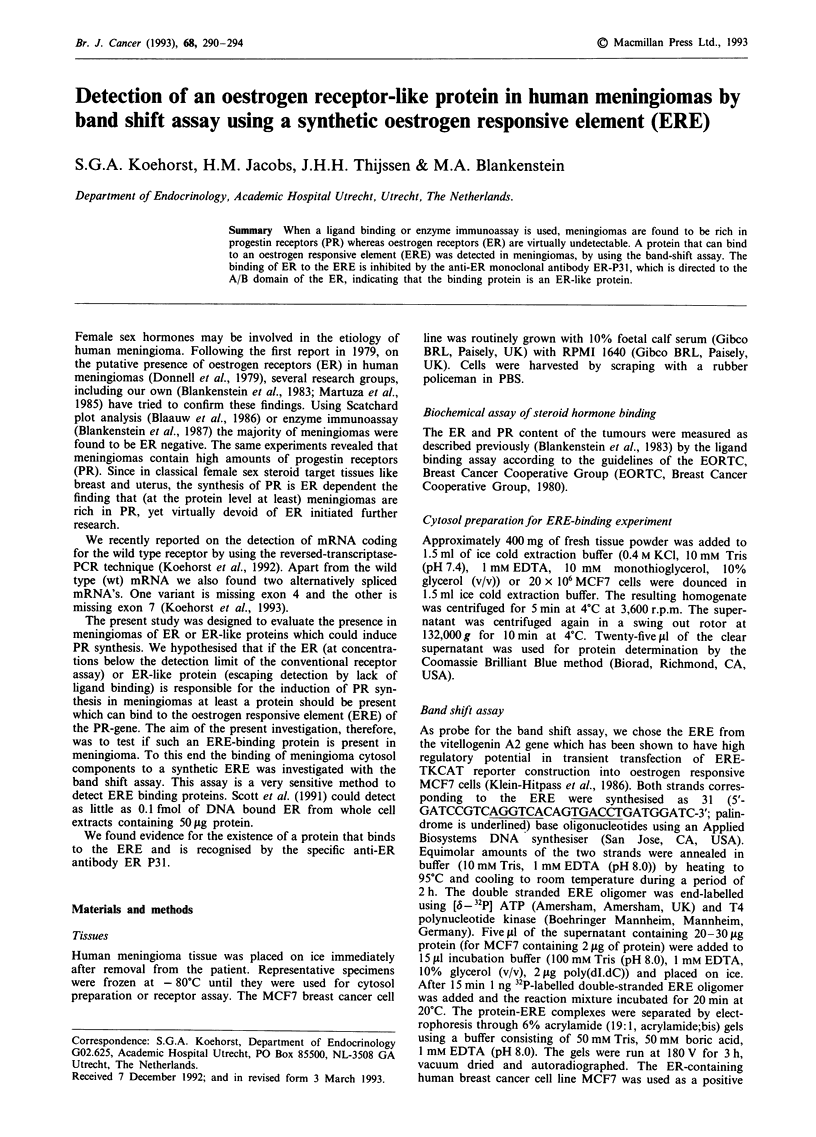

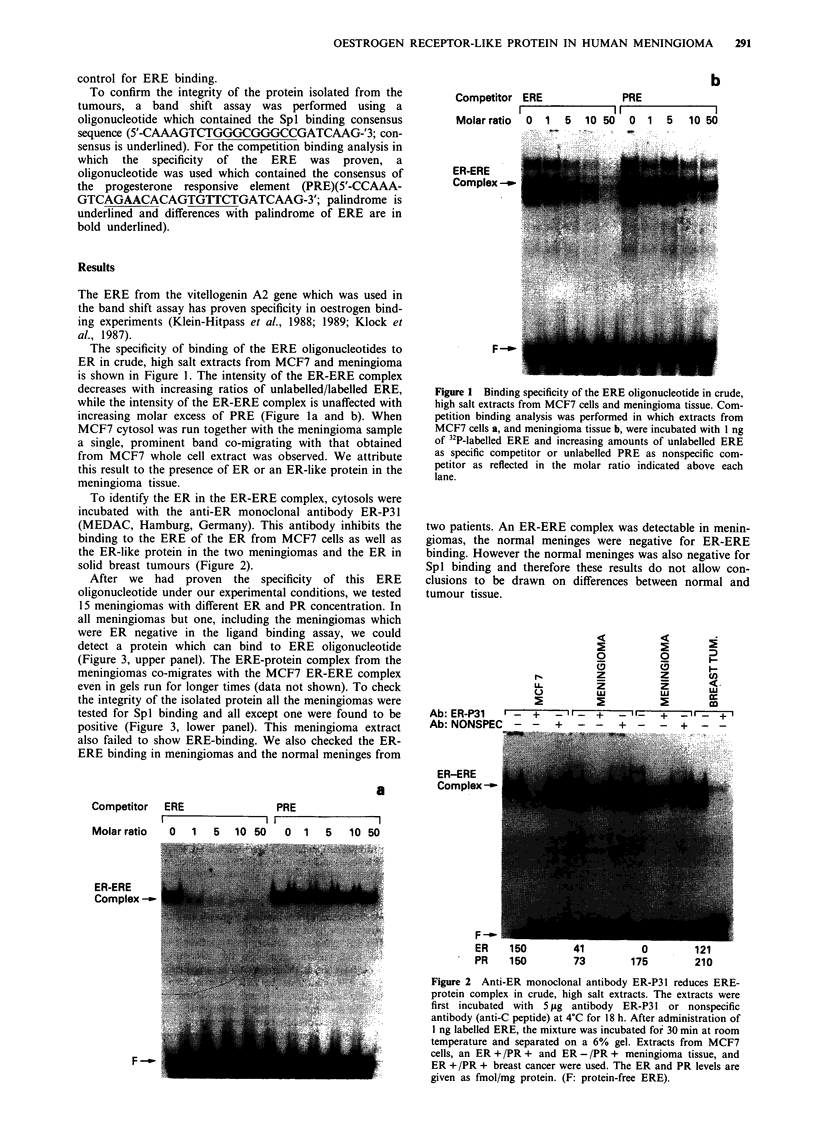

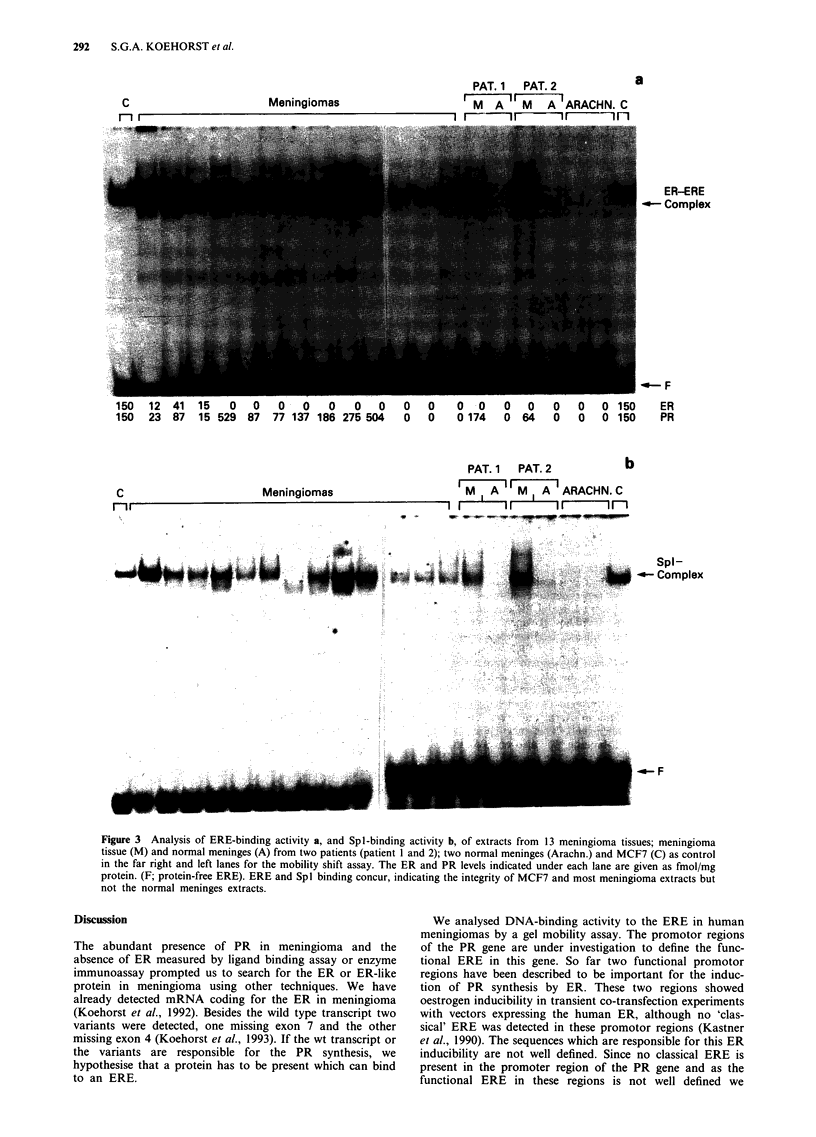

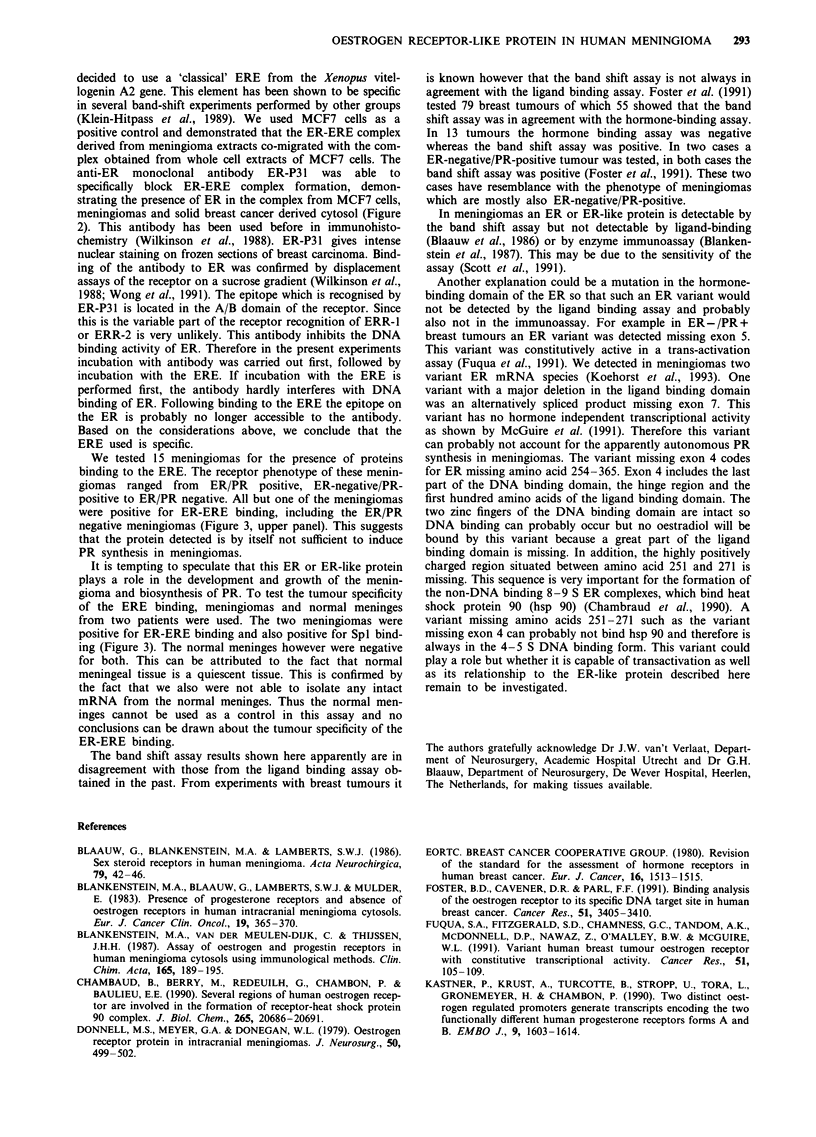

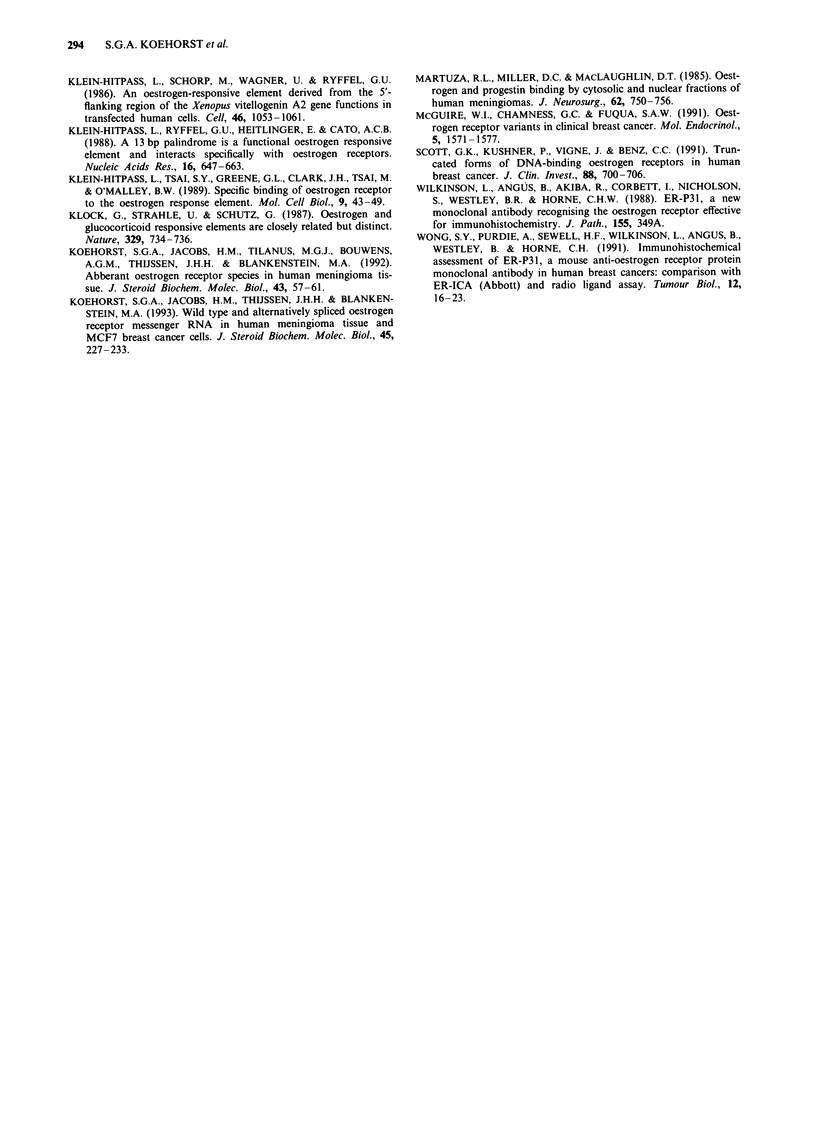


## References

[OCR_00472] Blaauw G., Blankenstein M. A., Lamberts S. W. (1986). Sex steroid receptors in human meningiomas.. Acta Neurochir (Wien).

[OCR_00477] Blankenstein M. A., Blaauw G., Lamberts S. W., Mulder E. (1983). Presence of progesterone receptors and absence of oestrogen receptors in human intracranial meningioma cytosols.. Eur J Cancer Clin Oncol.

[OCR_00483] Blankenstein M. A., van der Meulen-Dijk C., Thijssen J. H. (1987). Assay of oestrogen and progestin receptors in human meningioma cytosols using immunological methods.. Clin Chim Acta.

[OCR_00489] Chambraud B., Berry M., Redeuilh G., Chambon P., Baulieu E. E. (1990). Several regions of human estrogen receptor are involved in the formation of receptor-heat shock protein 90 complexes.. J Biol Chem.

[OCR_00495] Donnell M. S., Meyer G. A., Donegan W. L. (1979). Estrogen-receptor protein in intracranial meningiomas.. J Neurosurg.

[OCR_00505] Foster B. D., Cavener D. R., Parl F. F. (1991). Binding analysis of the estrogen receptor to its specific DNA target site in human breast cancer.. Cancer Res.

[OCR_00510] Fuqua S. A., Fitzgerald S. D., Chamness G. C., Tandon A. K., McDonnell D. P., Nawaz Z., O'Malley B. W., McGuire W. L. (1991). Variant human breast tumor estrogen receptor with constitutive transcriptional activity.. Cancer Res.

[OCR_00517] Kastner P., Krust A., Turcotte B., Stropp U., Tora L., Gronemeyer H., Chambon P. (1990). Two distinct estrogen-regulated promoters generate transcripts encoding the two functionally different human progesterone receptor forms A and B.. EMBO J.

[OCR_00532] Klein-Hitpass L., Ryffel G. U., Heitlinger E., Cato A. C. (1988). A 13 bp palindrome is a functional estrogen responsive element and interacts specifically with estrogen receptor.. Nucleic Acids Res.

[OCR_00526] Klein-Hitpass L., Schorpp M., Wagner U., Ryffel G. U. (1986). An estrogen-responsive element derived from the 5' flanking region of the Xenopus vitellogenin A2 gene functions in transfected human cells.. Cell.

[OCR_00538] Klein-Hitpass L., Tsai S. Y., Greene G. L., Clark J. H., Tsai M. J., O'Malley B. W. (1989). Specific binding of estrogen receptor to the estrogen response element.. Mol Cell Biol.

[OCR_00543] Klock G., Strähle U., Schütz G. (1987). Oestrogen and glucocorticoid responsive elements are closely related but distinct.. Nature.

[OCR_00556] Koehorst S. G., Jacobs H. M., Thijssen J. H., Blankenstein M. A. (1993). Wild type and alternatively spliced estrogen receptor messenger RNA in human meningioma tissue and MCF7 breast cancer cells.. J Steroid Biochem Mol Biol.

[OCR_00548] Koehorst S. G., Jacobs H. M., Tilanus M. G., Bouwens A. G., Thijssen J. H., Blankenstein M. A. (1992). Aberrant oestrogen receptor species in human meningioma tissue.. J Steroid Biochem Mol Biol.

[OCR_00561] Martuza R. L., Miller D. C., MacLaughlin D. T. (1985). Estrogen and progestin binding by cytosolic and nuclear fractions of human meningiomas.. J Neurosurg.

[OCR_00566] McGuire W. L., Chamness G. C., Fuqua S. A. (1991). Estrogen receptor variants in clinical breast cancer.. Mol Endocrinol.

[OCR_00571] Scott G. K., Kushner P., Vigne J. L., Benz C. C. (1991). Truncated forms of DNA-binding estrogen receptors in human breast cancer.. J Clin Invest.

[OCR_00582] Wong S. Y., Purdie A., Sewell H. F., Wilkinson L., Angus B., Westley B., Horne C. H. (1991). Immunohistochemical assessment of ER-P31, a mouse anti-oestrogen receptor protein monoclonal antibody in human breast cancers: comparison with ER-ICA (Abbott) and radioligand assays.. Tumour Biol.

